# Strategies for Management of Bile Duct Injury During Laparoscopic Cholecystectomy

**DOI:** 10.1155/DTE.7.55

**Published:** 2001

**Authors:** Tatsuya Aoki, Akihiko Tsuchida, Hitoshi Saito, Yuichi Nagakawa, Keiichi Kitamura, Yasuhisa Koyanagi

**Affiliations:** Department of Surgery Tokyo Medical University Tokyo Japan

## Abstract

We encountered 10 patients with bile duct injuries during laparoscopic cholecystectomy. Their causes were electrocautery in 2 patients, misjudgment in 2, mechanical injury in 3, aberrant bile duct in 2, and weakness of the bile duct wall in one. The sites of injury were cystic duct in 4 patients, common bile duct in 2, aberrant bile duct in 2, common hepatic duct in one, and common bile duct plus right hepatic duct in one. Treatments for the injuries discovered intraoperatively consisted of T-tube drainage above in 2 patients, re-ligation of the cystic duct in one, ligation of an aberrant bile duct in one, simple suture and T-tube in one, and choledochojejunostomy in one. In the remaining 4 patients discovered postoperatively, 2 were conservatively treated by endoscopic retrograde biliary drainage. The duration of hospitalization was 9–12 days in the 4 patients with simple suture or ligation, 10–21 days in 2 cases of bile drainage, and 34–43 days in 3 with T-tube drainage. The patient with choledochojejunostomy suffered repeated cholangitis, resulting in hepatic abscess with hospitalization for 6 months. Since laparoscopic surgery should be minimally invasive, meticulous attention is necessary before and during surgery to avoid bile duct injury.


*Diagnostic and Therapeutic Endoscopy*, Vol. 7, pp. 55–61
Reprints available directly from the publisher
Photocopying permitted by license only

© 2001 OPA (Overseas Publishers Association) N.V.
Published by license under
the Harwood Academic Publishers imprint,
part of Gordon and Breach Publishing,
a member of the Taylor & Francis Group.

### Strategies for Management of Bile Duct Injury during Laparoscopic Cholecystectomy

TATSUYA AOKI*, AKIHIKO TSUCHIDA, HITOSHI SAITO, YUICHI NAGAKAWA, KEIICHI KITAMURA and YASUHISA KOYANAGI

*Department of Surgery, Tokyo Medical University, Tokyo, Japan*

*(Received 14 August 2000; Revised 25 September 2000; In final form 17 October 2000)*

**We encountered 10 patients with bile duct injuries during laparoscopic cholecystectomy. Their causes were electrocautery in 2 patients, misjudgment in 2, mechanical injury in 3, aberrant bile duct in 2, and weakness of the bile duct wall in one. The sites of injury were cystic duct in 4 patients, common bile duct in 2, aberrant bile duct in 2, common hepatic duct in one, and common bile duct plus right hepatic duct in one. Treatments for the injuries discovered intraoperatively consisted of T-tube drainage above in 2 patients, re-ligation of the cystic duct in one, ligation of an aberrant bile duct in one, simple suture and T-tube in one, and choledochojejunostomy in one. In the remaining 4 patients discovered postoperatively, 2 were conservatively treated by endoscopic retrograde biliary drainage. The duration of hospitalization was 9–12 days in the 4 patients with simple suture or ligation, 10–21 days in 2 cases of bile drainage, and 34–43 days in 3 with T-tube drainage. The patient with choledochojejunostomy suffered repeated cholangitis, resulting in hepatic abscess with hospitalization for 6 months. Since laparoscopic surgery should be minimally invasive, meticulous attention is necessary before and during surgery to avoid bile duct injury.**

*Keywords*: Bile duct injury, Bile leakage, Laparoscopic cholecystectomy

### INTRODUCTION

Laparoscopic cholecystectomy (LC), first introduced in Europe, has been rapidly embraced worldwide as the procedure of choice for cholecystectomy [1–4]. Compared with open cholecystectomy, LC is associated with less postoperative wound pain, shorter hospitalization resulting in an early return to work, and a favorable cosmetic outcome [5,6]. Since LC is a minimally invasive technique, it must be safer than open laparotomy, however, intraoperative complications especially concerning bile duct injury during LC is still a matter of discussion. If such complications cannot be treated appropriately, severe infection or biliary stricture may result [7,8]. In the present study, we

* Corresponding author. Tel.: 03-3342-6111, ext. 5835. Fax: 03-3340-4575.

retrospectively reviewed causes and treatment of intraoperative bile duct injury and elucidated how to treat and prevent them.

### MATERIALS AND METHODS

Laparoscopic cholecystectomy was conducted in a total of 1094 patients at the Department of Surgery, Tokyo Medical University from December 1990 to December 1999. All patients, who had no history of upper abdominal operation, presence of advanced gallbladder carcinoma, and severe respiratory or circulatory problems, underwent laparoscopic surgery under general anesthesia. All patients received preoperative drip infusion cholangiography or magnetic resonance cholangiopancreatography for preoperative screening. For patients whose cystic ducts were not visualized by these examinations, endoscopic retrograde cholangiography (ERC) was performed to confirm the biliary tract passage. At the completion of surgery, a single drain was placed in the liver bed in all patients.

Bile duct injuries were classified according to the modified criteria of Strasberg *et al.* (Table I) [3]. Degrees of injury were classified into two groups: (1) minor injury consisting of a pinhole injury or injury of less than half the duct wall, and (2) major injury which indicated injury of more than half the bile duct wall. Based on these classifications, we investigated patient characteristics, causes, time of occurrence, treatment, and length of hospitalization.

### RESULTS

Of 1094 patients who underwent LC, 10 (0.9%) had bile duct injuries. They consisted of 5 men and 5 women, ranging in age from 23 to 74 years (mean, 50.3). The primary disease of these 10 patients was gallstones in 9 patients and gallbladder polyp in one. Six had a history of mild or severe cholecystitis, among which 2 patients developed inflammation due to ERC (Table II).

Causes of bile duct injuries were electrocautery burn in 2 patients, misjudgment of the biliary tract in 2, mechanical injury by clipping in 3, aberrant bile duct in 2, and weakness of the bile duct wall due to severe inflammation in one. In the 2 patients with aberrant bile duct injury, their ducts extended immediately dorsal to the cystic duct and thus were not seen on preoperative cholangiography or during surgery. In the other 8 patients, both the common bile duct and the cystic duct were clearly seen on preoperative cholangiography. Patient No. 2 had suffered a bile duct injury before the intraoperative cholangiography (Table II).

Types of bile duct injury were A2 in 4 patients, B in one, D in 4, and E5 in one. The site and degree of injury are shown in Table III. The injuries were diagnosed intraoperatively in 6 patients and postoperatively in 4.

Regarding treatment for the injuries discovered intraoperatively, all 6 were converted to open laparotomy and T-tube drainage was performed in 2 patients, religation of the cystic duct in one,

TABLE I Classification of bile duct injuries according to modified Strasberg's criteria

| Type | Characteristics |
|---|---|
| A1 | Leak from subvesical duct |
| A2 | Leak from cystic duct |
| B | Clipped and divided right or aberrant hepatic duct |
| C | Divided right hepatic or aberrant hepatic duct |
| D | Lateral injury to any bile duct except cystic duct |
| E1 | Common hepatic duct division more than 2 cm from the bifurcation |
| E2 | Common hepatic duct division less than 2 cm from the bifurcation |
| E3 | Common bile duct division at the bifurcation |
| E4 | Separate left and right hepatic duct strictures |
| E5 | Combined injury to the main duct at the bifurcation and right hepatic bile duct |

TABLE II Clinical features of the patients with bile duct injuries

| No. | Age | Gender | Diagnosis | Inflammation of GB | Adhesion | Cause of injury |
|---|---|---|---|---|---|---|
| 1 | 60 | F | GS | Absent | Absent | burn injury |
| 2 | 48 | M | GS | Present (severe) | Present (omentum) | misidentification |
| 3 | 26 | M | GS | Present (mild) | Present (omentum) | burn injury |
| 4 | 47 | M | GB polyp | Present* (mild) | Present (omentum) | mechanical injury |
| 5 | 62 | M | GS | Absent | Absent | mechanical injury |
| 6 | 23 | F | GS | Absent | Absent | aberrant bile duct |
| 7 | 63 | M | GS | Present* (severe) | Present (omentum) | misidentification |
| 8 | 74 | F | GS | Present (mild) | Present (omentum) | severe inflammation |
| 9 | 57 | F | GS | Absent | Absent | aberrant bile duct |
| 10 | 43 | F | GS | Present (mild) | Absent | mechanical injury |

GS: gallstones, GB: gallbladder.
*acute cholecystitis after endoscopic retrograde cholangiography.

TABLE III Characteristics and treatment of injuries

| No. | Time of diagnosis | Injury type | Site of injury | Treatment of injury | Duration of hospitalization after operation |
|---|---|---|---|---|---|
| 1 | After operation | A2, minor | CD | Re-ligation of CD | 10 days |
| 2 | Intraoperatively | D, major | CBD | T-tube drainage | 43 days |
| 3 | After operation | D, minor | CBD | Simple suture of injury | 12 days |
| 4 | After operation | A2, minor | CD | ERBD placement | 21 days |
| 5 | Intraoperatively | A2, minor | CD | Re-ligation of CD | 9 days |
| 6 | Intraoperatively | B, major | AD | Ligation of AD | 11 days |
| 7 | Intraoperatively | E5, major | CBD, RHD | Choledochojejunostomy | 6 months |
| 8 | Intraoperatively | D, minor | CHD | T-tube drainage | 34 days |
| 9 | Intraoperatively | D, minor | AD | Simple suture of injury<br>T-tube drainage | 41 days |
| 10 | After operation | A2, minor | CD | ERBD placement | 10 days |

CD: cystic duct, CBD: common hepatic duct, AD: aberrant hepatic duct, RHD: right hepatic duct, CHD: common hepatic duct, ERBD: endoscopic retrograde biliary drainage.

ligation of an aberrant bile duct in one, simple suture of the injury and T-tube drainage in one, and choledochojejunostomy in one. In patient No. 6, the aberrant bile duct was clipped and cut, resulting in the clip toward the liver side as being in inappropriate position due to the operative procedure and biliary leakage resulted. Thus, a laparotomy was done followed by taking images through the duct with a diameter of 2 mm, verifying the communication with the intrahepatic main bile duct, allowing the duct to be ligated. In the remaining 4 patients in whom biliary leakage developed postoperatively, ERC was performed in order to confirm the leakage sites. Two of the 4 patients were conservatively treated with endoscopic retrograde biliary drainage (ERBD). Since patient No. 1 was one of our earliest cases since beginning LC, laparotomy was done for confirmation and the cystic duct was religated. Since patient No. 3 showed minor leakage from the common bile duct, laparotomy was carried out, followed by simple suture of the injury (Table III).

Duration of hospitalization of the 10 patients was 9–12 days (mean, 10.5 days) in 4 patients with simple suture or ligation, 10–21 days (mean, 15.5 days) in 2 with ERBD placement, and 34–43 days in 3 with T-tube drainage. In addition, patient No. 7 stayed in hospital for 6 months after surgery (Table III). This case was caused by misjudgment of the biliary tract, which was due to severe cholecystitis and surrounding adhesion by preoperative ERC. Since the common bile duct and the right hepatic duct were cut and clipped during operation, choledochojejunostomy between the right, left hepatic duct and jejunum was performed. The patient developed repeat cholangitis due to the stenosis of anastomosis in the right hepatic duct, resulting in large abscess at the posterior segment of the liver. Therefore, he underwent the percutaneous transhepatic drainage and the administration of antibiotics for about 4 months (Fig. 1).

### DISCUSSION

Bile duct injury is one of the most frequent complications of cholecystectomy. The incidence in LC was 0.4–0.9%, which was higher than in patients who underwent open cholecystectomy (OC) [9–14]. This outcome is unsatisfactory for LC in terms of minimally invasive surgery and it must be necessary to decrease this iatrogenic injury.

There are three primary causes of bile duct injury. First, there are operator issues such as carelessness, rough operative procedure and misjudgment of the biliary tract. Of 10 patients in the present study, 7 injuries could be attributed to these causes; 3 were injured with the clipping instrument, 2 patients were burned because an electrocautery was used at the hepatocystic junction, resulting in accidental contact with the bile duct wall and a pinhole-sized injury. This could have been avoided by not using electrocautery around major biliary tracts such as the common bile duct and the common hepatic duct. In patients No. 2 and 7, the inflammatory wall thickening of the cystic duct made the boundary with the common bile duct indistinct. Consequently, the operators misunderstood the common bile duct for the cystic duct, which led to injury. Although there is the opinion that intraoperative cholangiography should be done routinely to avoid bile duct injury, this question remains controversial [15–17]. In patient No. 2, the injury was caused by misjudgment of the common bile duct for the cystic duct before intraoperative cholangiography to confirm

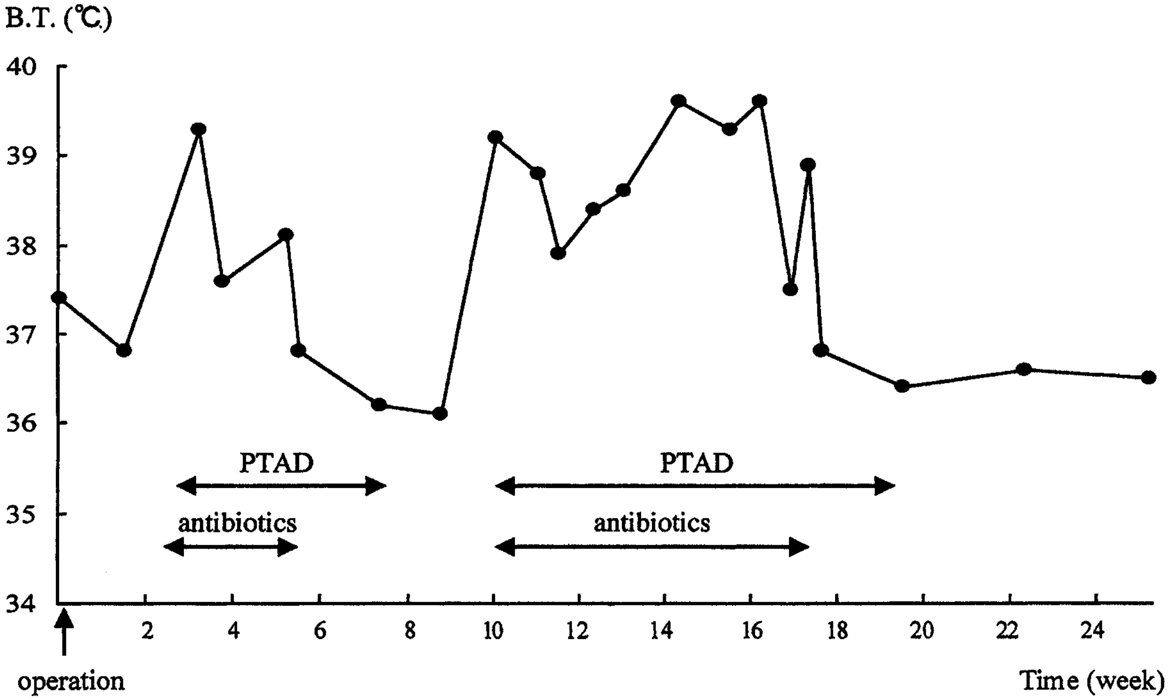

FIGURE 1 Clinical course of Patient No. 7. B.T.: body temperature, PTAD: percutaneous transhepatic abscess drainage.

the anatomical distribution of the biliary tract. Intraoperative cholangiography is designed to prevent bile duct injuries, but they frequently occur before insertion of the imaging catheter. To avoid this, as Inui *et al.* pointed out, it is essential to insert an imaging catheter through the cystic duct which is nearest the gallbladder or gallbladder neck [18].

The second reason for bile duct injury includes anatomical factors such as an anomalous junction of the bile duct or aberrant bile duct. In human autopsies, the frequency of aberrant bile duct is 12–28%. Most aberrant ducts arise from the right hepatic duct and drain most commonly into the common hepatic duct or cystic duct within 30 mm of the hepatocystic angle [19–22]. In previous reports 5–17% of patients with bile duct injury had an aberrant bile duct [23–25], while it was 20% in our study. In two of our patients the injuries were fortunately detected intraoperatively and were immediately treated appropriately. However, detection was occasionally delayed because the damaged aberrant bile duct could not be imaged under routine cholangiography. Although CT examination could easily diagnose the lesion in cases with dilation of intrahepatic bile duct [26], not all the cases could show this abnormality. Therefore, to prevent bile duct injury, it is essential to conduct preoperative cholangiography to identify the presence of an aberrant bile duct.

The third reason for bile duct injury involves pathologic factors such as severe inflammation around the gallbladder especially at the hepatocystic junction, adhesions from previous surgery, dislocation of bile duct, and Mirizzi syndrome. Patient No. 8 had a weakened bile duct wall due to inflammation, which contributed to a partial injury of the common hepatic duct despite a carefully executed operative procedure. Furthermore, severe inflammation of the biliary tract, especially around the hepatocystic junction, causes thickening of the cystic duct, resulting in an indistinct boundary between the common bile duct and common hepatic duct. This can cause the common bile duct to be mistaken for the cystic duct. Although there is a report that intraoperative ultrasonography can be useful to identify the cystic duct [27], we would like to emphasize that patients with severe inflammation should be converted to open laparotomy to avoid bile duct injury, which would be regarded as a kind of courageous withdrawal.

To prevent bile duct injury, two points should be noted regarding operative procedures. First, the gallbladder neck should be pulled in an appropriate direction in order to sufficiently open the triangle of Calot. This ensures better dissection of the cystic duct junction. Second, when dissecting the cystic duct, it is essential to initially expose the gallbladder neck followed by blunt dissection of the lesion from the exposed site toward the common bile duct. Strasberg *et al.* similarly pointed out that clipping of the cystic duct should be performed after the triangle of Calot, including the cystic artery, has been completely exposed [3]. Another report defined the best possible blunt dissection as exposure of the cystic duct by dissecting forceps, not by electrocautery [28]. During this dissection, there is frequently slight bleeding from vessels, but one should refrain from hemostasis with electrocautery or clips because the risk for bile duct injury increases.

If bile duct injury dose occur, the best strategy is to repair the injury intraoperatively if possible [29]. If the injury involves over half of a major biliary tract, T-tube drainage or hepaticoenterostomy may be indicated. In partial injury to the bile duct wall, the lesion is repaired using absorbable sutures and, in some cases, combined with bile duct drainage. When bile duct injury is overlooked at the initial operation, jaundice or peritonitis frequently occurs. Therefore, many of these patients are forced to undergo abdominal or bile duct drainage, followed by secondary biliary tract reconstruction after their systemic conditions improves. Thus, it is important to detect any bile duct injury during the initial operation. In cases of cystic duct injury, intraoperative re-clipping of the cystic duct should be performed, or, if it is impossible, a conversion to laparotomy is indicated. Past reports pointed out that cystic duct injuries rarely occurred during open cholecystectomy [13,28,29]. If the cystic duct injury should not be detected intraoperatively, ERBD placement is extremely effective. For example, Woods *et al.* reported that 8 of 17 patients with cystic duct injury showed improvement with ERBD [28]. On the other hand, Willis *et al.* reported that in patients with biliary leakage from a cystic or subvesical duct, suture of the injury by relaparoscopic operation or drainage was useful, and the mean hospitalization was 4 days, significantly less than our 15.5 days [29]. Therefore, depending on the condition of patients, these treatments should be considered.

Our patient No. 7 with E5 injury, which is the worst and most complicated one, developed repeated cholangitis with hepatic abscess, resulting in a 6-month hospitalization. Recently, there are some reports of simultaneous injury to the hepatic duct and hepatic artery, resulting in a hepatectomy [30,31]. As pointed out by Uenishi *et al.* [30], the patient with inadequate biliary reconstruction and recurrent cholangitis may undergo hepatectomy for the prevention of cholangitis. Since laparoscopic operations should be minimally invasive, meticulous attention is necessary before and during surgery to avoid these complications.

### References

[1] Reddick, E.J. and Olsen, D.O. Laparoscopic laser cholecystectomy. A comparison with mini-lap cholecystectomy. *Surg. Endosc.* 1989; **3**: 131–133.
[2] Dubois, F., Icard, P., Berthelot, G. *et al.* Coelioscopic cholecystectomy. Preliminary report of 36 cases. *Ann. Surg.* 1990; **211**: 60–62.
[3] Strasberg, S.M., Hertl, M. and Soper, N.J. An analysis of the problem of biliary injury during laparoscopic cholecystectomy. *J. Am. Coll. Sug.* 1995; **180**: 101–125.
[4] Jan, Y.Y., Chen, H.M., Wang, C.S. *et al.* Biliary complications during and after laparoscopic cholecystectomy. *Hepato-gastroenterol.* 1997; **44**: 370–375.
[5] Kane, R.L., Lurie, N., Borbas, C. *et al.* The outcomes of elective laparoscopic cholecystectomies. *J. Am. Coll. Surg.* 1995; **180**: 136–145.
[6] Soper, N., Stockman, P., Dunnegan, D. *et al.* Laparoscopic cholecystectomy: The new "gold standard"? *Arch. Surg.* 1992; **127**: 917–923.
[7] Manoukian, A.V., Schmalz, M.J., Geenen, J.E. *et al.* Endoscopic treatment of problems encountered after laparoscopic cholecystectomy. *Gastrointest. Endosc.* 1993; **39**: 9–14.
[8] Bezzi, M., Silecchia, G., Materia, A. *et al.* Complications after laparoscopic cholecystectomy. Coordinated radiologic, endoscopic, and surgical treatment. *Surg. Endosc.* 1995; **9**: 29–36.
[9] Deziel, D.J., Millikan, K.W., Economou, S.G. *et al.* Complications of laparoscopic cholecysytectomy: A national survey of 4,292 hospitals and analysis of 77,604 cases. *Am. J. Surg.* 1993; **165**: 9–14.
[10] Targarona, E.M., Marco, C., Balague, C. *et al.* How, when, and why bile duct injury occurs. *Surg. Endosc.* 1998; **12**: 322–326.
[11] Regöly-Mérei, J., Ihasz, M., Szeberin, Z. *et al.* Biliary tract complications in laparoscopic cholecystectomy. A multicenter study of 148 biliary tract injuries in 26,440 operations. *Surg. Endosc.* 1998; **12**: 294–300.
[12] Ooi, L.L.P.J., Goh, Y.C., Chew, S.P. *et al.* Bile duct injuries during laparoscopic cholecystectomy: A collective experience of four teaching hospitals and results of repair. *Aust. NZJ. Surg.* 1999; **69**: 844–846.
[13] Roslyn, J.J., Binns, G.S., Hughes, E.F.X. *et al.* Open cholecystectomy. *Ann. Surg.* 1993; **218**: 129–137.
[14] MacMahon, A.J., Fullarton, R.J., Baxter, J.N. *et al.* Bile duct injury and bile leakage in laparoscopic cholecystectomy. *Br. J. Surg.* 1995; **82**: 307–313.
[15] Fletcher, D.R. Operative cholangiogram at laparoscopic cholecys-tectomy. *Semin. Laparosc. Surg.* 1995; **2**: 11–117.
[16] Fletcher, D.R., Hobbs, M.S., Tan, P. *et al.* Complications of cholecystectomy: risks of the laparoscopic approach and protective effects of operative cholangiography: a population-based study. *Ann. Surg.* 1999; **229**: 449–457.
[17] Mirza, D.F., Narsimhan, K.L., Ferraz Neto, B.H. *et al.* Bile duct injury following laparoscopic cholecystectomy: Referral pattern and management. *Br. J. Surg.* 1997; **84**: 786–790.
[18] Inui, H., Kwon, A.H. and Kamiyama, Y. Management bile duct injury during and after laparoscopic cholecystectomy. *J. Hepatobiliary Pancreat. Surg.* 1998; **5**: 445–449.
[19] Mentzer, S.H. Anomalies of the bile ducts in man. *JAMA* 1929; **93**: 1273–1279.
[20] Williams, C. and Williams, A.M. Abnormalities of the bile ducts. *Ann. Surg.* 1955; **141**: 598–613.
[21] Healey, J.E. and Schroy, P.C. Anatomy of the biliary ducts within the human liver. *Arch. Surg.* 1953; **66**: 599–616.
[22] Moosman, D.A. Accessory bile duct. *Mich. Med.* 1964; **63**: 355–357.
[23] Van Sonnenberg, E., D'Agostino, H., Easter, D. *et al.* Complications of laparoscopic cholecystectomy: coordinated radiologic and surgical management in 21 patients. *Radiology* 1993; **188**: 399–404.
[24] Chartrand-Lefebvre, C., Dufresne, M., Lafortune, M. *et al.* Iatrogenic injury to the bile duct: a working classification for radiologists. *Radiology* 1994; **193**: 523–526.
[25] Suhocki, P.V. and Meyers, W.C. Injury to aberrant bile ducts during cholecystectomy: A common cause of diagnostic error and treatment delay. *Am. J. Roentgenol.* 1999; **172**: 955–959.
[26] Christensen, R., van Sonnenberg, E., Nemcek, A. Jr. *et al.* Inadvertent ligation of the aberrant right hepatic at cholecystectomy: radiologic diagnosis and therapy. *Radiology* 1992; **183**: 549–553.
[27] Tomonaga, T., Filipi, C.J., Lowham, A. *et al.* Laparoscopic intracorporeal ultrasound cystic duct length measurement: a new technique to prevent common bile duct injuries. *Surg. Endosc.* 1999; **13**: 183–185.

[28] Woods, M.S., Shellito, J.L., Santoscoy, G.S. *et al.* Cystic duct leaks in laparoscopic cholecystectomy. *Am. J. Surg.* 1994; **168**: 560–565.

[29] Aoki, T., Kimura, K., Tsuchida, A. *et al.* The clinical study for intraoperative injury of the biliary system. *J. Jpn. Soc. Clin. Surg.* 1992; **53**: 2363–2368.

[30] Uenishi, T., Hirohashi, K., Tanaka, H. *et al.* Right hepatic lobectomy for recurrent cholangitis after bile duct and heaptic artery injury during laparoscopic cholecystectomy: Report of a case. *Hepatogastroenterol.* 1999; **46**: 2296–2298.

[31] Nishio, H., Kamiya, J., Nagino, M. *et al.* Right hepatic lobectomy for bile duct injury associated with major vascular occlusion after laparoscopic cholecystectomy. *J. Hepatobiliary Pancreat. Surg.* 1999; **6**: 427–430.

